# Augmented Reality Navigation Can Achieve Accurate Coronal Component Alignment During Total Knee Arthroplasty

**DOI:** 10.7759/cureus.34607

**Published:** 2023-02-03

**Authors:** Kyle M Bennett, Andrew Griffith, Francesca Sasanelli, Isaac Park, Simon Talbot

**Affiliations:** 1 Department of Orthopaedic Surgery, Western Health, Melbourne, AUS; 2 Department of Orthopaedic Surgery, Melbourne Health, Melbourne, AUS

**Keywords:** orthopaedics, accuracy, augmented reality, computer assisted system, total knee arthroplasty

## Abstract

Background

Computer-navigated knee arthroplasty has been shown to improve accuracy over conventional instruments. The next generation of computer assistance is being developed using augmented reality. The accuracy of augmented reality navigation has not been established.

Methods

From April 2021 to October 2021, a prospective, consecutive series of 20 patients underwent total knee arthroplasty utilising an augmented reality-assisted navigation system (ARAN). The coronal and sagittal alignment of the femoral and tibial bone cuts was measured using the ARAN and the final position of the components was measured on postoperative CT scans. The absolute difference between the measurements was recorded to determine the accuracy of the ARAN.

Results

Two cases were excluded due to segmentation errors, leaving 18 cases for analysis. The ARAN produced a mean absolute error of 1.4°, 2.0°, 1.1° and 1.6° for the femoral coronal, femoral sagittal, tibial coronal and tibial sagittal alignments, respectively. No outliers (absolute error of >3°) were identified in femoral coronal or tibial coronal alignment measurements. Three outliers were identified in tibial sagittal alignment, with all cases demonstrating less tibial slope (by 3.1°, 3.3° and 4°). Five outliers were identified in femoral sagittal alignment and in all cases, the component was more extended (3.1°, 3.2°, 3.2°, 3.4° and 3.9°).

The mean operative time significantly decreased from the first nine augmented reality cases to the final nine cases by 11 minutes (p<0.05). There was no difference in the accuracy between the early and late ARAN cases.

Conclusion

Augmented reality navigation can achieve accurate alignment of total knee arthroplasty with a low rate of component malposition in the coronal plane. Acceptable and consistent accuracy can be achieved from the initial adoption of this technique, however, some sagittal outliers were identified and there is a clear learning curve with respect to operating time.

The level of evidence was IV.

## Introduction

Accurate and precise alignment of the mechanical components in total knee arthroplasty (TKA) is integral to achieving functional recovery and longevity of the prosthesis. Accurate alignment improves stability and load sharing and avoids prosthetic loosening or abnormal wear [1]. Computer-assisted TKA has been demonstrated to result in a greater proportion of accurate component positioning when compared to conventional techniques. Choong et al. identified a 17% greater rate of component malpositioning, defined as greater than 3° in the coronal plane, with conventional TKA as opposed to computer-assisted systems (CAS) [2]. Current trends towards personalised surgery have resulted in the increasing adoption of navigated and computer-assisted TKA. This has been in an attempt to achieve the reproducible alignment techniques required to attain subtle variations [3-5].

There is increasing interest in the development and use of immersive technologies, such as augmented reality, to achieve accurate component alignment. Augmented reality (AR) superimposes a computer-generated image onto a user’s view of the real world. In surgery, the use of devices such as smart glasses can create an enhanced overlay of cutting planes or axes, assisting surgeons to execute precise instrumentation [6,7]. Compared to CAS and conventional instrumentation, immersive technologies create an interface between the surgeon and computer-generated information, leading to improved surgical execution and offering a safe and cost-effective method for trainees to develop surgical techniques [1,6,8].

There are limited data on the accuracy and practicality of the use of AR assistance in TKA. Pre-clinical studies by Fallavollita et al. and later by Tsukada et al. have demonstrated the accuracy of their AR navigation systems in TKA [9,10]. Fallavollita et al., utilising a camera-augmented, mobile C-arm guide, initially identified a Pearson’s correlation of 0.979 between the C-arm and computer tomography (CT) data of mechanical axis deviation. Tsukada et al., utilising an intra-operative smartphone guide, later demonstrated a difference of less than 2° in internal/external rotation between values displayed on the smartphone technology and the resected bone measurements. They further reported a difference of less than 1° in varus/valgus and posterior slope angles in actual resection [9,10].

The Knee+ augmented reality navigation system for TKA (Pixee Medical Company, Besancon, France) assists surgeons with implant positioning through the wearing of smart glasses. The technology allows surgeons to visualise mechanical axes and provides real-time feedback on positioning and axes of resection. A clinical pilot study by Iacono et al. assessed the accuracy of this AR navigation system in five patients undergoing TKA. They demonstrated an error of less than 1° between the expected and resultant varus/valgus alignment of the femur and tibia, and an error of less than 2° in the flexion/extension of the femur and posterior tibial slope [11].

Our study aims to assess the accuracy of component positioning during TKA utilising the Knee+ AR system.

## Materials and methods

Patients

From April 2021 until October 2021, the Knee + (Pixee Medical Company, Besancon, France) system was used on a total of 20 patients. These cases were the first 20 cases performed by this surgeon (ST) using the augmented reality-assisted navigation (ARAN) system. Due to limitations in the availability of the system and intermittent COVID-19-related surgical restrictions, it was not a consecutive series. All patients underwent primary TKA and there were no exclusion criteria. All patients were informed about the use of the system and consented to allow data collection and analysis. Patients were included irrespective of the severity of deformity, age and body mass index (BMI). All patients received the Saiph Medial-Pivot prosthesis (Matortho, United Kingdom). The surgeries were all performed by the same orthopaedic surgeon (ST), who has performed over 800 replacements using this prosthesis and conventional instrumentation.

Demographic data and operative time were collected prospectively.

Preoperative CT scans were performed to guide a personalised surgical technique. The images consisted of 1.25 mm thick slices through the hip, knee and ankle (GE Optima 660 Brightspeed, 128-slice scanner). The resulting scans were segmented and analysed using the mediCAD® 3D Knee software (mediCAD Hectec GmbH, Altdorf/Landshut, Germany).

The coronal alignment of the femoral and tibial components was adjusted to compensate for preoperative hip-knee angle (HKA) and joint line obliquity (JLO). Alterations in HKA were restricted to between 0° to 3° varus. Femoral rotational alignment was based on the flexion-extension axis (FEA) of the femur based on the relative diameters of the posterior condyles and the surgical epicondylar axis (SEA) on the preoperative CT scan. Intraoperatively, it was based on the sulcus line of the trochlear groove [12]. Femoral sagittal alignment was adjusted from mechanical alignment based on the sagittal bow of the femur to avoid femoral notching and to allow downsizing of the femoral component. The tibial sagittal alignment was set at 7° of posterior slope in all cases, as per the recommended operative technique for this prosthesis.

The coronal alignment of the femoral and tibial components was measured at the component-cement interface, relative to the mechanical axes from the centre of the hip or ankle joint respectively. The sagittal alignment of the femoral component was measured from the femoral pegs. The sagittal alignment of the tibial component was measured at the superior surface of the component. All measurements were performed by two independent examiners, with the results of the two measurements averaged. Repeated measures were performed to assess intra-observer reliability. Surgical time was determined from skin incision to final skin closure.

Surgical technique

**Figure 1:**
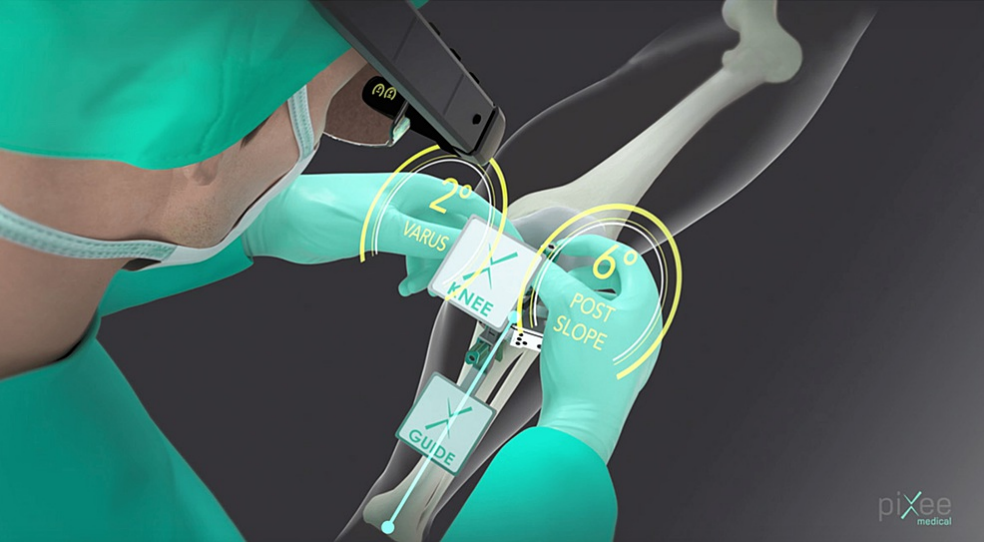
Schematic of intra-operative displayed angles and headset

**Figure 2:**
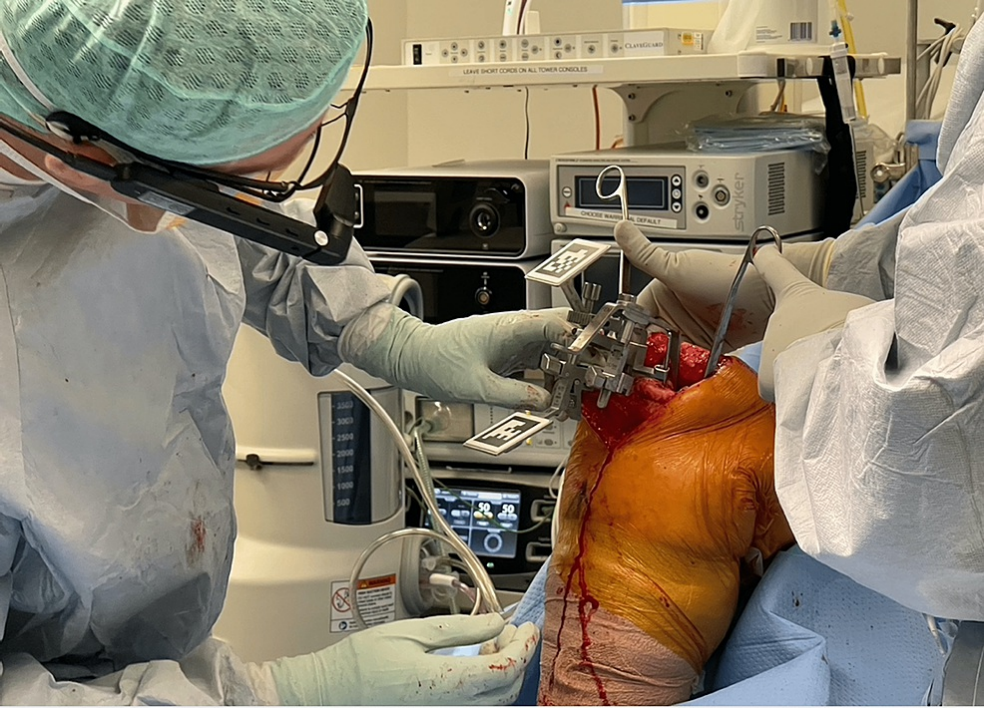
Article surgeon orientating metal jig with the AR system AR: augmented reality

The standard medial parapatellar approach was performed in all cases. The Knee+ system was used to align the distal femoral and proximal tibial cuts. The system utilises the Vuzix M400 Smart Glasses and quick response (QR) coded flags (Figure 1). For the femoral cut, the flag is attached to a metal jig, which is centred on a pin placed into the distal femur at the centre of the knee joint. A separate flag is temporarily bolted to the side of the table as a fixed reference point for the acquisition of the hip centre. The hip centre is obtained by rotation of the hip joint, whilst keeping the two flags within view of the smart glasses. Once the hip centre is obtained, the fixed reference flag is removed from the field of view. A cutting block with a further QR flag is then attached to the metal jig and dialled into the desired coronal and sagittal alignment (Figure 2). The cutting block is pinned, and then the metal jig and flags are removed. The distal bone cut is performed and then validated with a QR-flagged block relative to the cutting guide. The validated angles relative to the mechanical axis were recorded for comparison to the postoperative CT scans.

The tibial cut was performed in a similar manner with a metal jig pinned to the centre of the knee. The ankle joint was identified with a QR-flagged stylus to identify the medial and lateral malleoli as per the described technique. The cutting block was dialled to the required alignment and a cut was performed, without any recuts performed. Validation was performed relative to the navigated cutting block and the angles were recorded for comparison to the postoperative CT scans.

The components were all cemented into position. Care was taken to avoid flexion of the femoral component during insertion by forcing the introducer into extension. The knee was placed in extension until the cement had set.

The component position measured on the postoperative CT scans was compared to the angles of the bone cuts validated by the Knee+ system. The absolute difference was recorded. Outliers were determined as an absolute difference of greater than 3°. A priori power analysis determined that 18 cases were required to detect a 50% reduction in alignment outlier rates from previously published data on conventional techniques (a = 0.05 and b = 0.80) [13]. Statistical analysis was performed using StataCorp. 2021. Stata Statistical Software: Release 17. College Station, TX: StataCorp LLC.

Pearson’s correlation was used to assess both inter-observer and intra-observer agreements. Outlier rates, means and confidence intervals were calculated for the accuracy of data. Student's t-test was used for comparison of accuracy and operative time between early and late cases.

## Results

A total of 20 TKAs were completed using the Knee+ navigation system from Pixee Medical. Two scans in the series were unable to be measured due to technical errors with the formatting of the CT imaging, leading to the inability to perform accurate segmentation. Therefore, the analysis was performed on 18 cases.

Accuracy

For each TKA, the mean postoperative CT measurements of component position were compared to intraoperative measurements of the bone cuts recorded by the Knee+ system. The accuracy of the system was determined by the mean absolute error in femoral coronal, femoral flexion, tibial coronal and tibial slope angles between these two measurements. Across the 18 cases, ARAN technology produced mean absolute errors of 1.4°, 2.0°, 1.1° and 1.6° for femoral coronal, femoral flexion, tibial coronal and tibial slope angles respectively (Table 1).

**Table 1:** Summary of average absolute error and reliability

	Femoral valgus	Femoral flexion	Tibial varus	Tibial slope
Mean absolute error (° )	1.3	2.0	1.1	1.6
Number of outliers (>3° )	0	5	0	3
Inter-observer reliability	0.95	0.97	0.84	0.82
Intra-observer reliability	0.98	0.90	0.95	0.97

No outliers were identified in the femoral coronal or tibial coronal alignment absolute error groups. Three outliers (3.1°, 3.3° and 4°) were identified in the tibial sagittal alignment group, with all cases demonstrating less tibial slope than the validated bone cut using ARAN. Five outliers were identified in the femoral sagittal alignment group. All cases were more extended (3.1°, 3.2°, 3.2°, 3.4° and 3.9°) than the validated bone cuts using ARAN (Table 2).

**Table 2:** Intra-operative resection absolute error values

	Average of absolute error																	
Femoral valgus (° )	1.0	1.0	0.0	2.3	2.2	1.3	1.1	2.4	0.3	2.0	1.3	1.9	0.3	1.7	1.6	0.9	2.3	0.3
Femoral flexion (° )	2.0	3.1	2.2	3.9	1.3	1.0	1.9	3.1	0.8	0.9	2.3	1.8	0.3	1.9	3.3	0.3	2.9	3.4
Tibial varus (° )	0.9	2.4	1.6	2.1	0.5	1.4	0.5	0.9	1.4	2.0	1.5	1.5	0.3	0.3	1.4	1.4	0.2	0.3
Tibial slope (° )	0.1	0.3	2.2	1.4	1.4	0.0	2.6	0.6	1.7	2.7	1.0	0.2	3.3	2.2	3.1	1.1	4.0	0.9

Inter-observer and intra-observer agreements

There was a strong correlation in all measurements between the two observers, with correlations of 0.95, 0.97, 0.84 and 0.82 for femoral valgus, femoral flexion, tibial varus and tibial slope measurements, respectively. This agreement was established based on the raw error measurements instead of their absolute values, to adequately observe the agreement in signs of error measurements. Intra-observer agreement was similarly accurate, with correlations of 0.98, 0.90, 0.95 and 0.97, respectively, for femoral valgus, femoral flexion, tibial varus and tibial slope angle measurements (Table 1).

Learning curve

**Figure 3:**
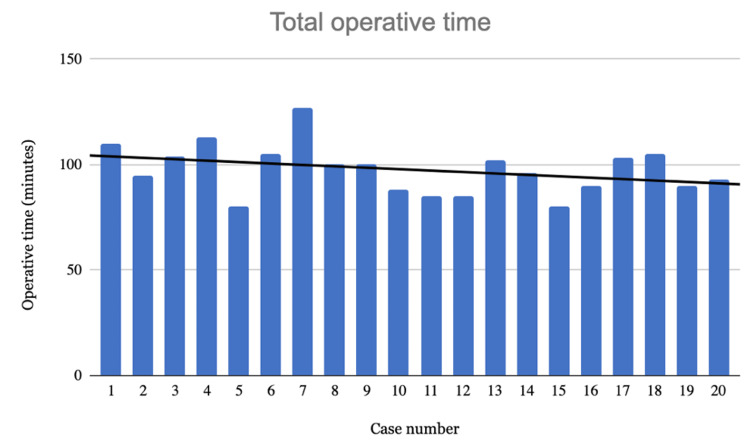
Graph of total operative time

**Figure 4:**
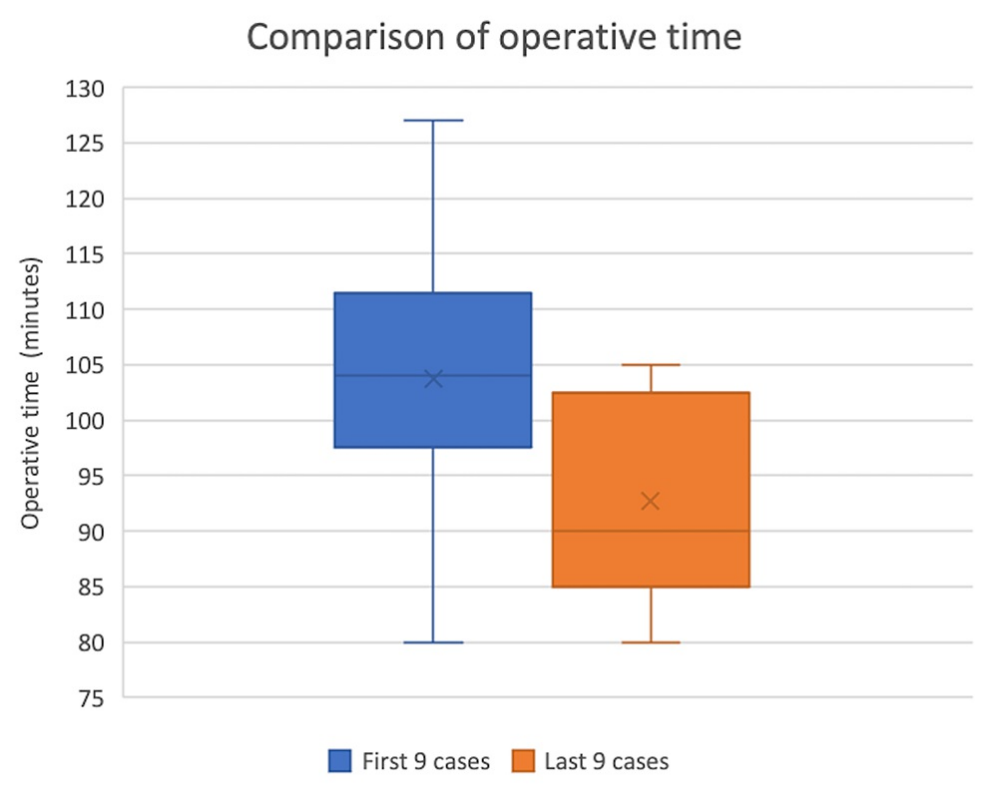
Comparison of operative time

**Figure 5:**
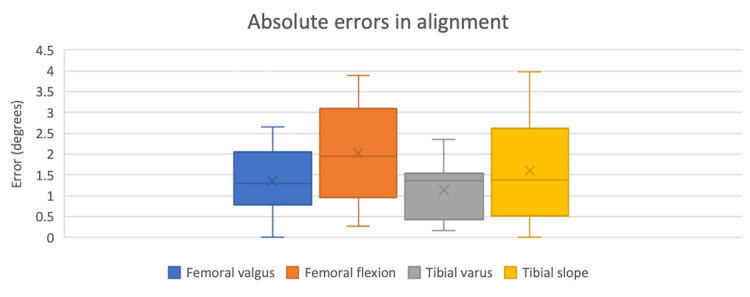
Comparison of absolute errors in alignment

The average operating time significantly decreased in the last nine cases by 11 minutes (t-value 2.11; p <0.05; CI (0.78, 21.44)), in comparison to the first nine cases (Figures 3, 4). There was no significant difference between the first nine and last nine cases in the absolute error of component position (Figure 5). There were no early complications identified and no significant BMI or age differences between the first and last nine patient cohorts (Table 3). All calculations were performed with STATA software.

**Table 3:** Comparison of first and last nine ARAN cases in mean absolute error and demographics ARAN: augmented reality-assisted navigation

	First 9 cases	Last 9 cases	P(T<=t) one tail
Femoral valgus (° )	1.3	1.4	0.45
Femoral flexion (° )	2.1	1.9	0.33
Tibial Varus (° )	1.3	1.0	0.17
Tibial slope (° )	1.1	2.1	0.05
Mean operative time (min)	104	93	0.023
Mean BMI	32.2	30.4	0.30

## Discussion

Our study aimed to demonstrate the reproducible accuracy of an ARAN system in 20 TKA patients, in comparison to their intra-operative observed measurements. Given that a priori power analysis determined 18 cases were required to detect a 50% reduction in alignment outlier rates, the loss of two patients due to segmentation errors was not deemed significant. To our knowledge at the time of writing, this is the second and largest clinical study of this size to analyse the accuracy of this system. The results suggest good coronal alignment was achieved, without outliers. The sagittal alignment results demonstrated less consistency with several outliers in both femoral and tibial alignment.

Through post-operative CT analysis, we were able to demonstrate a mean absolute error of 1.3°, 2.0°, 1.1° and 1.6° for measured angles of femoral coronal alignment, femoral flexion, tibial coronal alignment and tibial slope measurements, respectively, in comparison to intraoperative planned resection angles. These results are similar to those of Iacono et al. [11], who measured the results of five cases using the ARAN system and reported a 1° difference in coronal alignment of the femur and 2° in flexion/extension of the femur and posterior tibial slope. Outlier measurements of greater than 3° in our study were 0%, 28%, 0% and 17%, for femoral coronal alignment, femoral flexion, tibial coronal alignment and tibial slope, respectively. Comparably, a meta-analysis comparing CAS to conventional TKA of 3437 patients by Mason et al., found a CAS outlier rate (values greater than 3°) of 4.9%, 18.1%, 4% and 11.8% in femoral coronal alignment, femoral flexion, tibial coronal alignment and tibial slope measurements, respectively. This was in comparison to a rate of 16%, 26.1%, 11.1% and 17.8% for conventional techniques [13].

The sagittal outliers observed are likely to reflect the combination of errors that occur when: performing the bone cuts, inserting the component and measuring the component alignment on a CT scan. Kim et al., Bathis et al. and Plaskos et al. demonstrated a mean error of 0.7°, 1.4° and 1.3° discrepancy from their planned to their final resection angles, prior to prosthesis placement in the sagittal plane, respectively. Bathis et al. also noted a 24% (femoral cuts) and 14% (tibial cuts) higher incidence of resections greater than 1.5° difference in the sagittal plane, as opposed to the coronal plane. Kim et al. additionally demonstrated a further 1.7° difference after placement of the prosthesis and a statistically significant greater error (p<0.001) in the sagittal plane compared to the coronal [14-16].

The femoral flexion absolute error outliers observed in our study were all more than 3° extended than the planned position (Table 3). On average, the femoral flexion recorded on the postoperative CT scans was 2.0° extended relative to the validated bone cuts on the AR system. This systematic error may be explained by the operating surgeon’s deliberate technique of forcing the femoral component into extension during insertion and cementation. This was performed in order to reduce the risk of the prominence of the anterior flange of this particular prosthesis. In addition, a systematic error may have occurred from a difference between the centres of the femur and tibia identified intraoperatively with the use of the AR system and during the identification of these points on the postoperative CT scans. Further studies with larger numbers will be required to determine the true accuracy of ARAN in the sagittal plane.

Learning curve

The learning curve was analysed in two ways. With regard to surgical accuracy, there was no difference between the early and late cases. This suggested that accuracy can be achieved using the ARAN system from the early phase of adoption. Surgical time, however, was significantly longer (11 minutes) for the early cases compared to the late cases.

Limitations

The limitations of this study were its small sample size, lack of a control group and only radiographic, rather than patient-reported or observed outcomes. Larger comparative studies will be required to further assess the accuracy of the system.

## Conclusions

Augmented reality navigation can achieve accurate alignment of TKA with a low rate of component malposition in the coronal plane. Acceptable and consistent accuracy can be achieved from the initial adoption of this technique. However, some outliers in the sagittal plane were identified, and there is a clear learning curve with respect to operating time.
